# Formation of Copper Oxide Nanotextures on Porous Calcium Carbonate Templates for Water Treatment

**DOI:** 10.3390/molecules26196067

**Published:** 2021-10-07

**Authors:** Mahmud Diab, Karam Shreteh, Michael Volokh, Taleb Mokari

**Affiliations:** 1 Department of Chemistry, Ben-Gurion University of the Negev, Beer-Sheva 8410501, Israel; diabmah@post.bgu.ac.il (M.D.); shreteh@post.bgu.ac.il (K.S.); volokh@bgu.ac.il (M.V.); 2 Ilse Katz Institute for Nanoscale Science and Technology, Ben-Gurion University of the Negev, Beer-Sheva 8410501, Israel

**Keywords:** copper oxide, 3D structure, heavy metal, water purification, Sorites, microorganism template, pollutant decontamination

## Abstract

The necessity of providing clean water sources increases the demand to develop catalytic systems for water treatment. Good pollutants adsorbers are a key ingredient, and CuO is one of the candidate materials for this task. Among the different approaches for CuO synthesis, precipitation out of aqueous solutions is a leading candidate due to the facile synthesis, high yield, sustainability, and the reported shape control by adjustment of the counter anions. We harness this effect to investigate the formation of copper oxide-based 3D structures. Specifically, the counter anion (chloride, nitrate, and acetate) affects the formation of copper-based hydroxides and the final structure following their conversion into copper oxide nanostructures over porous templates. The formation of a 3D structure is obtained when copper chloride or nitrate reacts with a *Sorites* scaffold (marine-based calcium carbonate template) without external hydroxide addition. The transformation into copper oxides occurs after calcination or reduction of the obtained Cu_2_(OH)_3_X (X = Cl^−^ or NO_3_^−^) while preserving the porous morphology. Finally, the formed *Sorites*@CuO structure is examined for water treatment to remove heavy metal cations and degrade organic contaminant molecules.

## 1. Introduction

In spite of the tremendous progress in technology, science, and medicine, nowadays, millions of people still do not have access to a clean water source [1]. For this reason, several approaches were developed for water purification, relying on activated carbon, reverse osmosis, ion exchange, distillation, and others [2,3,4,5,6,7,8]. Furthermore, a lot of effort is invested in seeking the best candidate materials. Metal oxides, such as TiO_2_, Fe_2_O_3_, MnO_2_, CeO_2_, MgO, CuO, and others, were extensively used in various water purification processes due to their properties, specifically, high sorption capacity, high stability, abundance, low cost, and low toxicity [9,10,11,12,13,14]. All purification techniques benefit from using materials with a large surface area-to-volume ratio. In such cases, a significant fraction of their active sites is exposed, which, in turn, facilitates contaminants’ adsorption (and possibly their decomposition in a secondary step). Developing materials with hierarchical structures can markedly improve the purification performance. A three-dimensional (3D) design increases the surface-to-volume ratio significantly (vs. a 2D design) and grants the possibility of interaction between the pollution (the adsorbate) and the active material (the adsorbent, acting as the filter).

Recently, CuO has attracted particular attention of the water purification community because of its antimicrobial properties, capability for driving dye degradation reactions, high sorption capacity for heavy metal ions (for instance, As(III) and As(V), Pb(II), Cd(II), and Cr(VI) [13,14,15,16]. A ceramic membrane that incorporates a CuO active material rejects more than 97.14% of Pb (II) and 91.44% of Cr (VI) [17,18]. Moreover, CuO nanowires show promising activity in oil-water separation [19]. Several reported works evaluated the CuO activity for methylene blue (MB) oxidation in the presence of hydrogen peroxide in aqueous solutions [20,21]. CuO is also photocatalytically active, allowing harnessing a radical-based mechanism for organic contaminants degradation and antibacterial performance. For example, the degradation of methyl orange was completed using CuO catalyst via photogenerated ·OH radicals [22]. Additionally, hydrogen peroxide (H_2_O_2_) was used, in the presence of CuO, to increase the radicals’ production rate by more than one order of magnitude [23]. This combination was successfully examined in the degradation of a brominated flame retardant [24] and to improve the antibacterial performance of CuO versus (vs.) *E. coli* [23]. Furthermore, the antibacterial activity of CuO vs. *E. coli* and *S. aureus* was found to be size-dependent—the smaller the CuO particles are, the higher their antibacterial activity becomes [25].

The progress in the synthesis of CuO manifests in the variety of shapes and sizes that have been reported via different approaches. For example, nanoparticles (sonochemical [26], precipitation [27]), nanorods (hydrothermal) [24,28], 3D structures (templates as mesoporous silica KIT-6 [29], copper foam [30], or anodic aluminum oxide [31]), thin films (chemical vapor deposition) [32], and close-packed films (combination of thermal decomposition and oxidation) [33] were reported. However, there is a lingering need to develop methods that tackle specific disadvantages of the previously mentioned approaches. For instance, when two separate steps are utilized (e.g., first formation and then assembly of particles), it complicates integration into devices. The fabrication of 3D structures either uses small templates that must be assembled on a supporting substrate, resulting in the loss of a high percentage of its total surface area, or the templates are difficult to remove (while preserving the morphology).

Herein, we demonstrate a facile method to form a porous structure of CuO via a two-step reaction. First, a copper-based hydroxide 3D structure is prepared by reacting a copper salt with a calcium carbonate template without hydroxide salt addition (the chosen template is *Sorites*—a marine calcareous foraminiferal shell of few mm diameter [34]). Subsequently, the obtained structure undergoes a reduction or calcination process to form *Sorites*@Cu_2_O or *Sorites*@CuO, respectively. *Sorites* consists mainly of CaCO_3_, which is easy to remove using a mild acid. In addition to the net-like structure, the *Sorites* has internal tunnels that connect its holes. We believe that these tunnels may confine the reactants, which increases the collision probability of the reactant molecules, and in turn, improves the performance of the active material [34,35,36].

Furthermore, it was reported that the nature of the counter anion of the copper complex (such as Cl^−^, SO_4_^2−^, NO_3_^−^, and CH_3_COO^−^) has a direct influence on the size, shape, and crystal structure of the hydroxide product [37,38,39,40]. However, to the best of our knowledge, there is no report discussing the effect of the counter anion on the formation of a 3D structure. Therefore, three different Cu(II) salts—CuCl_2_, Cu(NO_3_)_2_, and Cu(ac)_2_—were used to elucidate their role in the formation of the 3D structure. Finally, we examine the performance of the prepared CuO 3D structures for water treatment, specifically for heavy metal ions removal and for the degradation of contaminant organic dyes as methylene blue.

## 2. Results and Discussion

**Figure 1:**
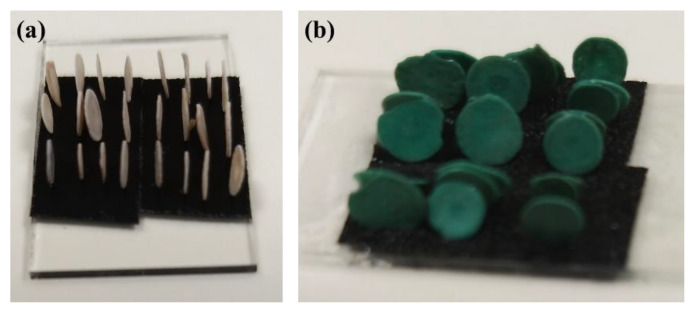
Optical images of the *Sorites* before (**a**) and after (**b**) reacting with CuCl_2_ to form a 3D structure of copper-based hydroxide.

To form the hereinafter reported 3D porous structures, *Sorites* scaffolds of marine origin were cleaned and assembled perpendicularly on a substrate using carbon tape, as shown in Figure 1a. The substrate was then immersed into the reaction vessel containing the Cu(II) salt to form the copper hydroxide-based coating (Figure 1b). See more details in the Materials and Methods (Section 3.2 and Section 3.3).

### 2.1. Formation of 3D Structures of Sorites@Copper-Based Hydroxide Using Various Copper Salts

**Figure 2:**
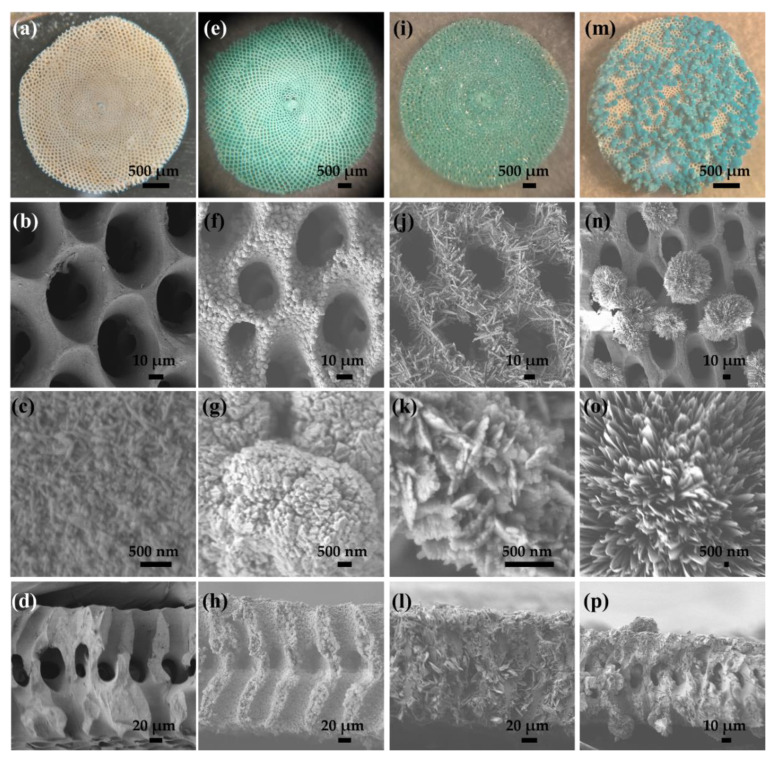
Optical and scanning electron microscopy (SEM) images of the *Sorites* 3D structure before (left column, **a**–**d**) and after reacting with three different copper salts (the subsequent columns from left to right): CuCl_2_ (**e**–**h**), Cu(NO_3_)_2_ (**i**–**l**), and Cu(ac)_2_ (**m**–**p**). (**a**,**e**,**i**,**m**) optical images, SEM top-view images, and SEM cross-section images (**d**,**h**,**l**,**p**).

Figure 2 shows optical and scanning electron microscopy (SEM) images of the *Sorites* before (a–d) and after reacting with CuCl_2_ (e–h), Cu(NO_3_)_2_ (i–l), and Cu(ac)_2_ (m–p) at 120 °C for 45 min (details in the experimental section). The optical images of the *Sorites* structures dramatically change before and after reacting with the copper salt, from white to turquoise, respectively. Furthermore, the visual images confirm that the morphology of the *Sorites* structure is well-kept when *Sorites* react with CuCl_2_ and Cu(NO_3_)_2_ to form the 3D structure of copper-based hydroxide. The SEM images present the homogeneity of the formed shell and show that the shell layer consists of particles with a typical size of a few hundred nm. Furthermore, the SEM images of the cross-section view (Figure 2d,h,l) show evidence of coating the internal surface of the *Sorites* structure. When Cu(ac)_2_ was used, the template morphology was preserved, but an aggregation of copper acetate was formed and precipitated on the surface of the *Sorites*, as portrayed in the SEM images in Figure 2n–p.

The difference in the quality of the formed shell layer between the three copper salts can be attributed to the acidity of the growth solution. CuCl_2_ and Cu(NO_3_)_2_ dissolve in water to give an acidic growth solution (pH ≈ 4), while Cu(ac)_2_ provides a milder one (pH ≈ 6, due to hydrolysis of the acetate conjugate base). The acidity of the growth solution plays an important role, particularly in our case, when *Sorites* is used (as this template consists of CaCO_3_). The solubility of CaCO_3_ significantly increases with increasing solution acidity. The *K*_sp_ of CaCO_3_ (calcite phase) at 25 °C is 3.36 × 10^−9^, and the p*K*_a_ of H_2_CO_3_ is 6.35 [41]. The pH of the growth solution must be low enough to diminish the homogenous nucleation of a copper hydroxide precipitate in the solution and high enough to retain the 3D structure of the CaCO_3_ template. We found that the shell formation was better when the pH of the growth solution was about four. This result is attributed to the improvement in the dissolution rate of the CaCO_3_ surface in solutions that contain the chloride or the nitrate counter anions rather than the acetate, as discussed below (Equations (1)–(3)). Furthermore, we believe that the dissolution of the *Sorites’* surface increases the surface roughness, which, in turn, acts as a nucleation site for the copper hydroxide shell formation.

**Figure 3:**
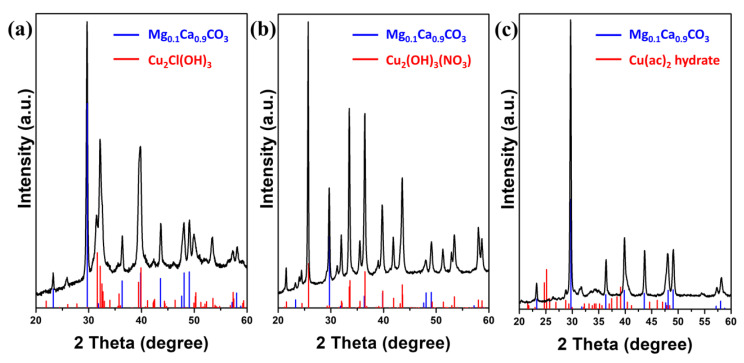
X-ray diffraction patterns of the 3D structures after reacting *Sorites* with different copper salts. (**a**) *Sorites*@Cu_2_Cl(OH)_3_, (**b**) *Sorites*@Cu_2_(OH)_3_(NO_3_), and (**c**) *Sorites* decorated with Cu(ac)_2_ hydrate. The blue and red bars in the XRD panels correspond to the rhombohedral Mg_0.1_Ca_0.9_CO_3_ and the coated material, respectively.

To further investigate the effect of the counter anion, structural characterization was conducted by X-ray diffraction, as shown in Figure 3. In all three cases, the obtained diffraction patterns match a rhombohedral Mg_0.1_Ca_0.9_CO_3_ (JCPDS card no. 071-1663) and the formation of additional diffraction signals, which can be assigned to the *Sorites* and copper-based hydroxide, respectively. Figure 3a,b shows that the reaction between *Sorites* and CuCl_2_ or Cu(NO_3_)_2_ forms an orthorhombic copper(II) hydroxychloride (Cu_2_Cl(OH)_3_, JCPDS card no. 071-2027), or monoclinic copper(II) hydroxynitrate (Cu_2_(OH)_3_NO_3_, JCPDS card no. 075-1779), respectively. When the *Sorites* reacts with Cu(ac)_2_, no copper hydroxide is found in the final product, and the diffraction pattern of Cu(ac)_2_ hydrate (JCPDS card no. 010-0756) best matches the obtained XRD pattern, as presented in Figure 3c, though this diffraction is weak and may indicate only partial crystallinity.

Usually, to form a Cu(OH)_3_X (X = Cl^−^, NO_3_^−^, CH_3_COO^−^, and others), CuX_2_ reacts with a hydroxide source (such as NaOH or Mg(OH)_2_) [38,40]. However, in the reported approach, Cu(OH)_3_Cl and Cu(OH)_3_NO_3_ are formed by reacting the CuX_2_ with the *Sorites* (the template) without adding a hydroxide salt. We think that the *Sorites* template is also responsible for the formation of the required hydroxide anions through the proposed mechanism:

(1) $${\mathrm{CaCO}}_{3}\left(s\right)+H_{3}O^{+}\left(\mathrm{aq}\right)⇌{\mathrm{Ca}}^{2+}\left(\mathrm{aq}\right)+{\mathrm{HCO}}_{3}^{-}\left(\mathrm{aq}\right)+H_{2}O\left(l\right)$$

(2) $${\mathrm{HCO}}_{3}^{-}\left(\mathrm{aq}\right)+H_{2}O\left(\mathrm{aq}\right)⇌{\mathrm{OH}}^{-}\left(\mathrm{aq}\right)+H_{2}{\mathrm{CO}}_{3}\left(\mathrm{aq}\right)$$

(3) $$2{\mathrm{Cu}}^{2+}\left(\mathrm{aq}\right)+3{\mathrm{OH}}^{-}\left(\mathrm{aq}\right)+X^{-}\to {\mathrm{Cu}}_{2}{\left(\mathrm{OH}\right)}_{3}X\left(s\right)$$

In the first stage, due to the acidity of the growth solution (pH ≈ 4), the CaCO_3_ surface dissolves to form HCO_3_^−^(aq). Subsequently, the bicarbonate undergoes a hydrolysis reaction to produce OH^−^(aq), and in the final step, the copper salt reacts with the produced hydroxide to form the Cu_2_(OH)_3_X. The proposed mechanism explains well the uniformity of the formed shell. Notably, the shell coats well the internal surface of the template, as presented in the cross-section images in Figure 2h,l, when CuCl_2_ and Cu(NO_3_)_2_ were used. The OH^−^(aq) is formed close to the template’s surface, where Cu_2_(OH)_3_X precipitates to form the shell layer. The fact that using Cu(ac)_2_ does not form a Cu_2_(OH)_3_(CH_3_COO) and the coating is not uniform, as presented in Figure 2m–p, supports the proposed mechanism as the growth solution is not acidic enough to drive Reaction 1.

### 2.2. Using Sorites@Cu_2_Cl(OH)_3_ as a Prototype to Further Investigate the Coating Process and Their Conversion to Sorites@CuO

The thickness of the Cu_2_Cl(OH)_3_ shell layer was controlled by either changing the reaction time or using a different concentration of CuCl_2_, as presented in Figure S1 (Supplementary Material). For example, increasing the reaction time from 45 min to 7 h leads to an increase in the shell thickness, from 1–2 µm to several µm (5–10 µm). In comparison, keeping the same reaction time and reducing the CuCl_2_ amount to 25 mg results in the formation of a shell with a thickness of ca. 500 nm.

**Figure 4:**
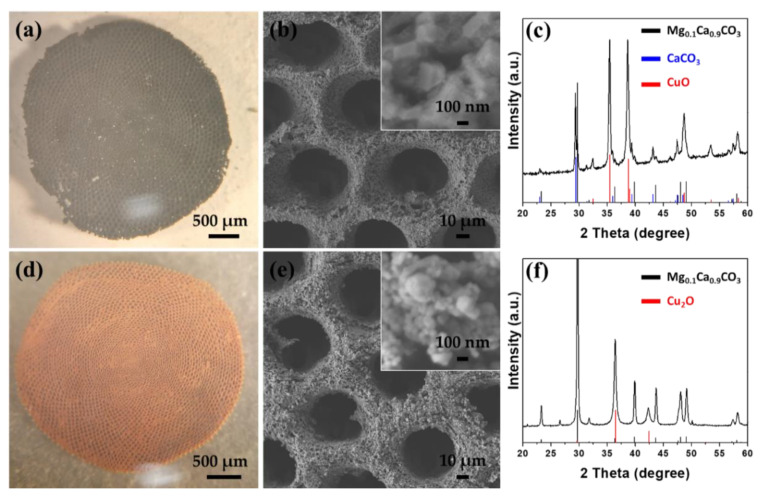
Structural characterization of the 3D structure after converting the hydroxide shell to an oxide. (**a**,**d**) The optical images, (**b**,**e**) SEM images, and (**c**,**f**) XRD patterns of *Sorites*@CuO and *Sorites*@Cu_2_O, respectively. The black, blue, and red stick patterns in the XRD panels correspond to the rhombohedral Mg_0.1_Ca_0.9_CO_3_, rhombohedral CaCO_3_, and the coated materials, respectively.

Most often, to produce metal oxides in aqueous solutions, two-step syntheses are used. Usually, the direct product, the metal-based hydroxide, undergoes a conversion process to form the metal oxide [23,24,25,27,28,42]. Figure 4 shows the structural characterization of the 3D structure after the effective conversion of Cu_2_Cl(OH)_3_ to copper oxide (CuO or Cu_2_O) by reduction or calcination, respectively. *Sorites*@CuO was formed after annealing the *Sorites*@Cu_2_Cl(OH)_3_ at 400 °C for 2 h under air. *Sorites*@Cu_2_O was formed after reacting the *Sorites*@Cu_2_Cl(OH)_3_ with hydrazine for several minutes. The 3D morphology was preserved after the conversion processes, as shown in the optical and SEM images in Figure 4a,b,d,e. The color change (from turquoise to black or red-orange) and the obtained XRD pattern endorse the successful transformation of the orthorhombic Cu_2_Cl(OH)_3_ to the respective copper oxide phase. According to a previous work [35], heating *Sorites* at high temperatures results in the formation of an additional phase (CaCO_3_) alongside the rhombohedral Mg_0.1_Ca_0.9_CO_3_. Figure 4c shows that after calcination, the obtained diffraction matches with rhombohedral Mg_0.1_Ca_0.9_CO_3_ (JCPDS card no. 071-1663), rhombohedral CaCO_3_ (JCPDS card no. 005-0586), and monoclinic copper(I) oxide (CuO, JCPDS card no. 045-0937). After a reduction process, the achieved XRD pattern is assigned to rhombohedral Mg_0.1_Ca_0.9_CO_3_ and cubic copper(II) oxide (Cu_2_O, JCPDS card no. 065-3288), as shown in Figure 4f. Furthermore, the absence of Cu_2_Cl(OH)_3_ diffraction signal confirms a complete structural conversion.

### 2.3. Potential Application of Sorites@CuO in the Removal of Heavy Metal Ions and Degradation of an Organic Model Dye Molecule

We demonstrate the activity of the proposed system in this work by removing heavy metal cations (Pb^2+^ and Cd^2+^) from water and in the degradation of methylene blue (MB). In our previous work, we have shown the contribution of the porous 3D structure (*Sorites*-based) relative to their 2D counterparts [36]. Based on these results, we have chosen to deposit the active copper oxides on the porous structures. Reacting the *Sorites*@CuO with a metal-containing solution leads to a significant reduction in the concentration of metal cations. The concentrations of Pb^2+^ (Cd^2+^) dropped from 122 ppm (98 ppm) to 4.8 ppm (0.21 ppm), which corresponds to a purification of 96% (99.7%).

It is important to point out that the mass of copper oxide is around 17% of the total mass of the formed 3D structure. Thus, the adsorption capacity of the active material (CuO) for Pb^2+^ and Cd^2+^ is 78 mg/g and 66 mg/g, respectively. These values are lower than the reported values for Pb^2+^ (115–125) and Cd^2+^ (192) [9,16]. This difference can be attributed to the relatively thick coating layer of CuO.

To rule out the formation of metal hydroxide as the reason for the removal of Pb^2+^ and Cd^2+^, we made sure that the pH of the heavy metal cation solution is not sufficient to lead to precipitation of the metal hydroxides (Equations (4) and (5)). At room temperature, precipitation ensues at a pH value calculated using Equation (6).

(4) $$\mathrm{Pb}{\left(\mathrm{OH}\right)}_{2}\left(s\right)⇌{\mathrm{Pb}}^{2+}\left(\mathrm{aq}\right)+2{\mathrm{OH}}^{-}\left(\mathrm{aq}\right)$$

(5) $$\mathrm{Cd}{\left(\mathrm{OH}\right)}_{2}\left(s\right)⇌{\mathrm{Cd}}^{2+}\left(\mathrm{aq}\right)+2{\mathrm{OH}}^{-}\left(\mathrm{aq}\right)$$

(6) $$\mathrm{pH}=14-\log\frac{\sqrt{\left[M^{2+}\right]}}{\sqrt{K_{\mathrm{sp}}}}$$

The solubility of lead and cadmium hydroxide is 1 × 10^−16^ and 7.2 × 10^−15^, respectively [10,41]. Thus, to precipitate a Pb(OH)_2_ or Cd(OH)_2_ starting with [Pb^2+^] = 0.59 µM or [Cd^2+^] = 0.87 µM, a pH value of 7.6 or 8.5 is required, respectively. However, in the described case, the pH of the heavy metal solutions was 6, which confirms that the removal of the cation is attributed to the action of CuO.

**Figure 5:**
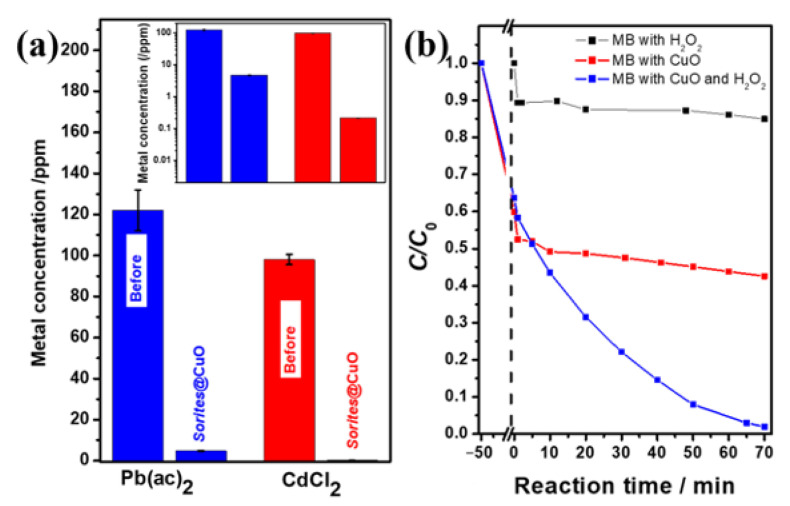
The performance of *Sorites*@CuO in water purification processes. (**a**) Different concentrations of Pb^2+^ (blue bars) and Cd^2+^ (red bars) were removed from the water by the *Sorites*@CuO 3D structure. The inset shows the concentrations of the Pb^2+^ and Cd^2+^ before and after removal using a logarithmic scale. (**b**) MB dye molecule degradation using *Sorites*@CuO. Complete degradation (blue) is achieved when the dye is mixed with both H_2_O_2_ and *Sorites*@CuO; two control conditions: degradation experiment (black) where the dye is mixed with H_2_O_2_ only; adsorption experiment (red) where the dye is mixed with *Sorites*@CuO only.

Figure 5b presents a model organic dye degradation reaction in an aqueous solution containing MB molecules using H_2_O_2_ only (black), *Sorites*@CuO only (red), and *Sorites*@CuO in the presence of H_2_O_2_ (blue). The addition of H_2_O_2_ to the MB solution reduces the concentration of the dye by 15%, and a plateau is observed around *C*/*C*_0_ = 0.85. When a *Sorites*@CuO catalyst was used, a 50 min incubation with MB solution was carried out to reach an adsorption–desorption equilibrium before monitoring the degradation reaction. After 50 min of incubation, the MB concentration reached about half of its initial value. Then, a slow decrease in concentration was observed, reaching a relative concentration of 0.42 after 70 min (degradation of ~20% relative to *t* = 0). The addition of H_2_O_2_ to an MB solution that contains the *Sorites*@CuO, after reaching a similar adsorption–desorption equilibrium, allowed the successful degradation of more than 98% of the initial MB after 70 min (blue line). This significant improvement can be assigned to the reaction between MB and the formed radicals. Previous work suggests that the result of H_2_O_2_ addition to a solution that contains CuO is the formation of several radicals such as ·OH (for oxidation) and ·O_2_ (for reduction) [25]. Furthermore, Figure S2 shows that the *Sorites*@CuO with H_2_O_2_ enables the reduction of the concentration of other dyes, specifically rhodamines—Rh6G and RhB—to 11% and 36% of their respective initial concentrations.

## 3. Materials and Methods

### 3.1. Materials

Cadmium (II) chloride (CdCl_2_, 99+%), copper (II) acetate (Cu(ac)_2_, 99%), copper (II) chloride (CuCl_2_, 98%), copper (II) nitrate trihydrate (Cu(NO_3_)_2_·3H_2_O, 99.5%), copper (II) sulfate (CuSO_4_, 98%), and lead (II) acetate trihydrate (Pb(ac)_2_·3H_2_O, 99.999%) were purchased from Strem Chemicals (Newburyport, MA, USA). Hydrazine monohydrate (98%), hydrogen peroxide (H_2_O_2_, 30% wt.), methylene blue (MB), and rhodamine 6G (Rh6G, 95%) were purchased from Sigma-Aldrich (St. Louis, MI, USA). Sodium hypochlorite (11–14% wt. available chlorine, assay result (iodometric titration) of 11.8% wt.) and rhodamine B (RhB) were purchased from Alfa Aesar (Heysham, Lancashire, UK). Hydrochloric acid (HCl, 32% wt.) was purchased from Bio-Lab (Jerusalem, Israel). All reagents were used as received without further purification. Deionized (DI) water was purified using a Millipore Direct-Q system (18.2 MΩ cm resistivity).

### 3.2. Purification and Pretreatment of Sorites Bio-Template

**Figure 6:**
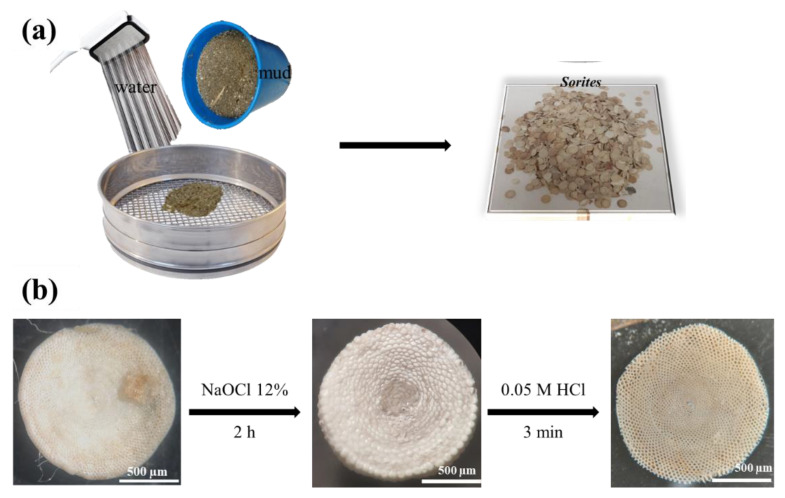
Purification and pretreatment processes of *Sorites*: (**a**) sifting and washing, (**b**) chemical pretreatment.

The *Sorites* bio-templates were collected from the red sea (Interuniversity Institute for Marine Sciences of Eilat) and were separated from other species by a sifting and washing process, as shown in Figure 6a. Figure 6b shows the surface cleaning and the etching process. The *Sorites* species were immersed in a sodium hypochlorite solution (12% wt. OCl^−^) for two hours. Finally, the top layer of the *Sorites* template was removed by soaking in 0.05 M HCl solution for 3 min.

### 3.3. Formation of a Copper Oxide Porous 3D Structure

The *Sorites* species (30 mg) were placed perpendicular to the substrate using carbon tape, as shown in Figure 1a. The substrate was diagonally placed inside a PTFE-lined autoclave (25 mL container), which contains 7 mL of 0.21 M copper (II) solution (Cu(ac)_2_, CuCl_2_, or Cu(NO_3_)_2_). Subsequently, the autoclave was heated to 120 °C for 45 min. The products (*Sorites* coated hydroxide-based material, Figure 1b) were washed with water and dried at 60 °C. The *Sorites*@Cu_2_Cl(OH)_3_ was annealed at 400 °C for 2 h under air (heating rate of 10 °C min^−1^ from room temperature to the target temperature) to form the *Sorites*@CuO. *Sorites*@Cu_2_O was obtained by adding 100 µL of hydrazine solution to the copper-based hydroxide and reacting for 5 min.

### 3.4. Removal of Heavy Metal Ions

Forty mL of contaminated solution was prepared using DI water and Pb(ac)_2_·3H_2_O (16 mg) or CdCl_2_ (7 mg). Atomic absorption spectroscopy (AAS, Perkin Elmer Analyst 400, PerkinElmer, Inc., Waltham, MA, USA) was used to measure the concentration of 10 mL of Pb^2+^ and Cd^2+^ solutions before and after reacting with *Sorites*@CuO 3D structure. The concentration of the prepared heavy metal solution was 122 ppm of Pb^2+^ (Figure 5a, blue bar) and 98 ppm of Cd^2+^ (Figure 5a, red bar). *Sorites*@CuO (100 mg) was shaken with 10 mL of Pb^2+^ or Cd^2+^ solutions for one hour at room temperature. The pH of the prepared solution was low (pH 6) to prevent the possible precipitation of Pb(OH)_2_ or Cd(OH)_2_.

The AAS measurements of concentrations (both before and after the removal process) were repeated three times to calculate the standard deviation. Before each measurement, a calibration curve was performed using standard solutions, and each sample was filtered with a 0.22 μm PVDF syringe filter.

### 3.5. Dye Degradation

Stock solutions of dye molecules (MB, RhB, or Rh6G) were prepared by dissolving the powdered dyes in DI water. The concentrations were adjusted such that the O.D. at 664 nm was ~1 for MB, O.D. at 554 nm was ~1.45 for RhB, and O.D. at 526 nm was ~1.12 for Rh6G. *Sorites*@CuO (60 mg) was incubated with 6 mL of the respective dye solution for 50 min under dark conditions at room temperature to achieve an adsorption-desorption equilibrium between the dye and the surface of the porous 3D structure. When H_2_O_2_ was used, the incubation was followed by the addition of 340 µL of H_2_O_2_ solution (30% wt.). UV−vis absorbance values were used to examine the change in the dye absorbance at *λ*_max_ (MB—664 nm, RhB—554 nm, and Rh6G—526 nm) and calculation of *C*/*C*_0_.

### 3.6. Structural Characterization

Scanning electron microscopy (SEM) was performed using a JEOL JSM-7400F high-resolution SEM system with a cold field emission gun, which was operated at 3.5 kV. Energy-dispersive X-ray spectroscopy (EDS) analysis was conducted using an SEM-coupled Thermo Scientific Noran SIX system at an accelerating voltage of 15.0 kV. Phase analysis of the samples was carried out using the X-ray diffraction (XRD) method. The data was collected on an Empyrean powder diffractometer (Malvern Panalytical, Worcestershire, UK) equipped with a position-sensitive X’Celerator detector using Cu Kα radiation (*λ* = 1.5418 Å), operated at 40 kV and 30 mA. Optical absorbance measurements were made using a Cary 5000 UV−vis−NIR spectrophotometer using standard 10 mm quartz cuvettes.

## 4. Conclusions

A simple approach for the formation of porous 3D structures of copper oxides (CuO, Cu_2_O) and copper-based hydroxides using the template method was demonstrated. It is found that the counter anion plays an essential role both in the formation of the 3D structure and in the formation of the copper-based hydroxide coating. The *Sorites* scaffold has two functions: (i) serving as the template for the structure and (ii) forming a local basic environment, which allows the precipitation of copper hydroxide throughout the porous structure. To allow the local hydroxide precipitation, the initial pH of the growth solution has to be low enough (i.e., 4 when chloride or nitrate counterions were used) to allow the partial dissolution of the CaCO_3_ template, releasing free hydroxide anions. These, in turn, allow the formation of the copper hydroxide throughout the porous 3D structure of the template, resulting in its coating. The copper oxide (CuO or Cu_2_O) 3D structures were formed in a subsequent step (by calcination or reduction, respectively). Finally, the prepared CuO structure allows the removal of more than 96% of heavy metal cations and degradation of more than 98% of an organic dye molecule.

## Data Availability

The data presented in this study are available on request from the corresponding author.

## Supplementary Materials

The following are available online: Figure S1: SEM images of *Sorites*@Cu_2_Cl(OH)_3_ obtained after reacting the *Sorites* (a,b) with 25 mg of CuCl_2_ at 120 °C for 45 min and (c,d) with 200 mg of CuCl_2_ at 120 °C for 7 h. Figure S2: Degradation of (a) RhB and (b) Rh6G using different catalysts. The vertical black dashed lines show the time of degradation initiation (*t* = 0). Black: the dyes were mixed with H_2_O_2_ only; red: the dyes were mixed with the *Sorites*@CuO only; blue: the dyes were mixed with both H_2_O_2_ and *Sorites*@CuO.

## Sample Availability

Samples of the compounds are available from the authors.
