# Supplementary material for: Formation of Copper Oxide Nanotextures on Porous Calcium Carbonate Templates for Water Treatment
Source: Molecules. 2021 Oct 7;26(19):6067. doi: 10.3390/molecules26196067 (PMC8512094; doi:10.3390/molecules26196067)
Supplement: Supplementary file 1 [file molecules-26-06067-s001.zip › molecules-1399355-supplementary.pdf]

Supporting Information for

Formation of Copper Oxide Nanotextures on Porous Calcium Carbonate Templates for Water Treatment

Mahmud Diab [1,†], Karam Shreteh [1], Michael Volokh [1] and Taleb Mokari [1,2,*]

1 Department of Chemistry, Ben-Gurion University of the Negev, Beer-Sheva 8410501, Israel; diabmah@post.bgu.ac.il (M.D.); shreteh@post.bgu.ac.il (K.S.); volokh@bgu.ac.il (M.V.)
2 Ilse Katz Institute for Nanoscale Science and Technology, Ben-Gurion University of the Negev, Beer-Sheva 8410501, Israel
* Correspondence: mokari@bgu.ac.il
† Current address: The Institute of Applied Research, The Galilee Society, Shefa-Amr 20200, Israel.

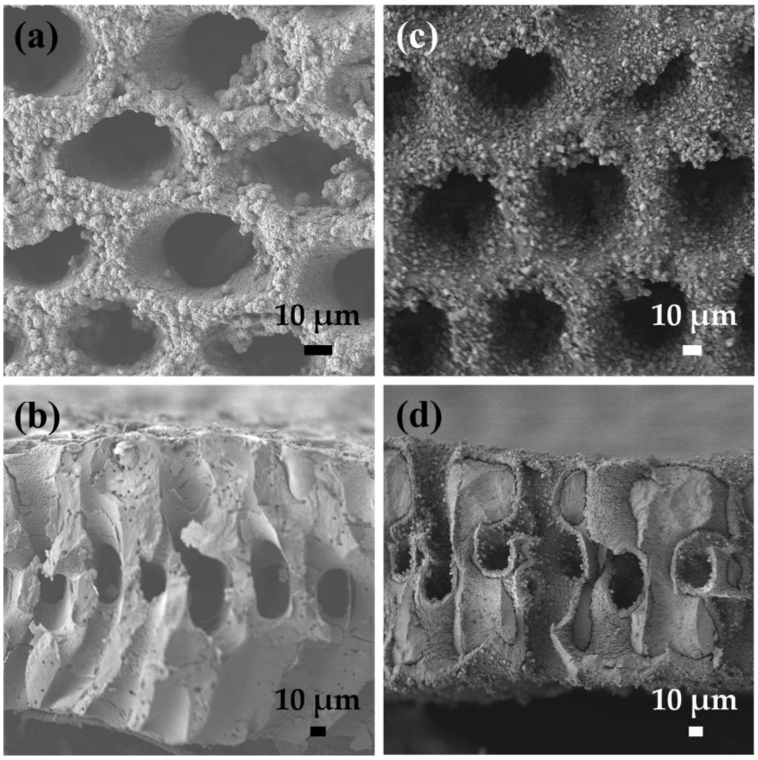

Figure S1. SEM images of *Sorites*@$Cu_2Cl(OH)_3$, obtained after reacting the *Sorites* (**a**,**b**) with 25 mg of $CuCl_2$ at 120 °C for 45 min, and (c,d) with 200 mg of $CuCl_2$ at 120 °C for 7 h.

The measured thickness of the coating layer (Figure S1b) when using the standard reaction conditions but a smaller copper salt amount (25 mg instead of 200 mg) results in 0.5 µm thickness (compared to 1–2 µm, see Figure 2h).

The measured thickness of the coating layer (Figure S1d) when using the standard reaction conditions but a longer reaction time (7 h instead of 45 min) results in a thicker 5–10 µm layer (compared to 1–2 µm, see Figure 2h).

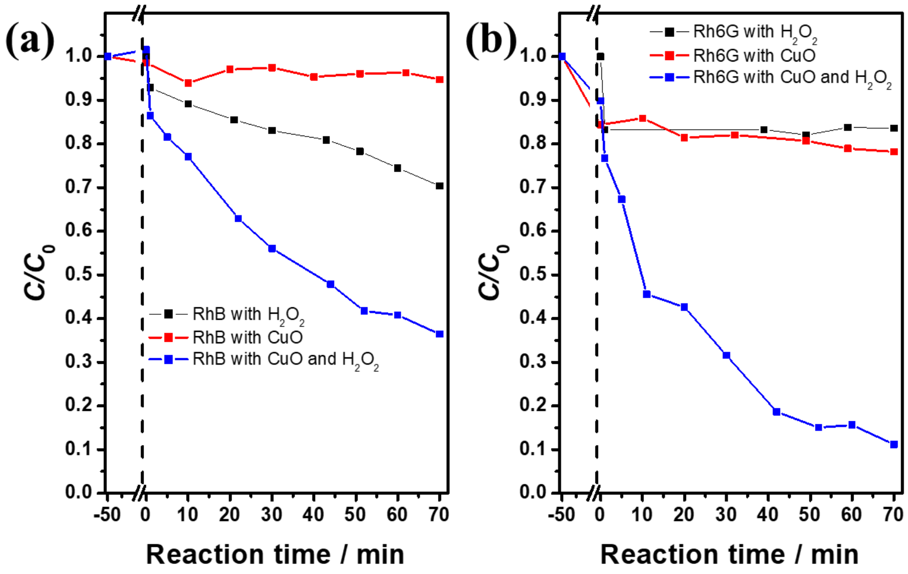

Figure S2. Degradation of (**a**) RhB and (**b**) Rh6G using different catalysts. The vertical black dashed lines show the time of degradation initiation ($t = 0$). Black: the dyes were mixed with H_2O_2 only; red: the dyes were mixed with the *Sorites*@CuO only; blue: the dyes were mixed with both H_2O_2 and *Sorites*@CuO.
